# Effects of Exercise Type and Sertraline on Anxiety and Social Behavior in a Female Rat Model of PTSD

**DOI:** 10.1002/brb3.71528

**Published:** 2026-07-23

**Authors:** Sarieh Ebrahimiasl, Farzam Sheikhzadeh Hesari, Mohammad Reza Khansari, Afshin Dana, Khadijeh Imani Minaabad

**Affiliations:** ^1^ Department of Clinical Psychology, Faculty of Psychology and Education Sciences Azad University, Central Tehran Branch Tehran Iran; ^2^ Department of Animal Biology, Faculty of Natural Sciences University of Tabriz Tabriz Iran; ^3^ Department of Veterinary Basic Sciences, Science and Research Branch Azad University Tehran Iran

**Keywords:** anxiety, exercise, Post‐traumatic Stress Disorder, sertraline, social behavior

## Abstract

**Background:**

Post‐traumatic stress disorder (PTSD) often co‐occurs with anxiety and social interaction difficulties, particularly in females. This study examined the effects of voluntary and involuntary exercise, combined with sertraline, on PTSD‐like symptoms, anxiety, and social behavior in female rats.

**Methods**

**::**

PTSD‐like symptoms were induced using the Single Prolonged Stress (SPS) model, which involved sequential exposure to restraint stress (2 h), forced swimming (20 min), and ether anesthesia (2–3 min), followed by a 7‐day incubation period. A 10‐min restraint was then applied as an additional stress event. Afterward, the rats underwent voluntary wheel running or treadmill exercise (5 days per week), with or without sertraline treatment (10 mg/kg/day) for 4 weeks. Behavioral assessments were conducted using the Elevated Plus Maze (EPM) and the Three‐Chamber Social Test (3‐CST). One day after behavioral testing, estrous cycles were evaluated using vaginal smears, followed by corticosterone analysis in blood samples the next day.

**Results:**

Involuntary exercise and sertraline significantly reduced anxiety‐like behavior, with involuntary exercise showing a stronger effect than voluntary exercise. Involuntary exercise also improved sociability and social novelty, while sertraline enhanced social novelty. Notably, sertraline reduced corticosterone levels, with the greatest effect observed when combined with involuntary exercise.

**Conclusion:**

These findings suggest that combining involuntary exercise with pharmacological treatment may provide synergistic benefits for alleviating anxiety‐like behaviors and stress responses in a PTSD‐like rat model.

## Graphical abstract

Time scale for procedures of three different treatments, including voluntary exercise, involuntary exercise, and sertraline administration.

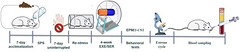

**Abbreviations**
3‐CST: three‐chamber social test.
EPM: elevated plus maze
PTSD: post‐traumatic stress disorder
SPS: single‐prolonged stress
SSRIs: selective serotonin reuptake inhibitors

## 1 Introduction

Post‐Traumatic Stress Disorder (PTSD) is a multifaceted mental health condition stemming from exposure to traumatic events, including physical or emotional abuse, accidents, or natural disasters (Sadock 2007). It is characterized by symptoms including intense fear, intrusive memories, hyperarousal, and avoidance behaviors, significantly impairing quality of life and social functioning (Weiss 2007; Diagnostic 2013). Epidemiological evidence indicates a higher prevalence of PTSD and anxiety disorders in women compared to men (Haering, Meyer et al. 2024). Moreover, females exhibit distinct stress‐related behavioral and neuroendocrine responses, partly influenced by ovarian hormones (Ter Horst et al. 2009).

Pharmacotherapy and behavioral interventions are pivotal in PTSD treatment (Williamson and Greenberg 2019). Currently, selective serotonin reuptake inhibitors (SSRIs) represent primary pharmacological treatments for PTSD (Bisson et al. 2020). Sertraline is an approved, first‐line SSRI for the treatment of PTSD and is also widely used in the management of other anxiety disorders, including generalized anxiety disorder, panic disorder, and social anxiety disorder (Bandelow et al. 2012). However, the effectiveness of pharmacological treatments can differ among individuals (Vaswani, Linda et al. 2003), prompting the proposal of physical exercise as a complementary non‐pharmacological approach, shown to reduce anxiety (Kandola and Stubbs 2020) and improve mood (Kramer 2020). Moreover, recent studies have investigated the combined effects of SSRI pharmacotherapy and exercise, showing that integrating these approaches can produce greater reductions in PTSD, anxiety, and depressive behaviors, including improvements in fear extinction (Khameslo et al. 2023; Shafia et al. 2023).

In addition, previous studies have shown that different forms of exercise may offer distinct mental health benefits (Miller, Gonçalves‐Bradley et al. 2020; Yu et al. 2022). In rodent studies, physical exercise is typically categorized as either voluntary, such as self‐paced wheel running, which provides psychological benefits through autonomy and motivation (Greenwood and Fleshner 2019), or involuntary, like treadmill running, which ensures consistent activity but lacks control for the subject. Studying different types of exercise in animal models can reveal how autonomy and motivation influence engagement, as voluntary exercise allows self‐paced activity while involuntary exercise limits control and represents a highly structured and disciplined regimen. This distinction helps to understand how exercise type affects psychological and physiological outcomes in PTSD.

The estrous cycle in female rodents, analogous to the menstrual cycle in humans, consists of several phases marked by specific hormonal changes (Ajayi and Akhigbe 2020). Corticosterone, the primary glucocorticoid hormone in rodents, is released in response to stress and plays a crucial role in regulating metabolism, immune function, and brain activity (Godoy et al. 2018; Joëls et al. 2018). In rodent models of PTSD, prolonged stress leads to long‐term increases in corticosterone levels, which are associated with enhanced anxiety‐like behaviors (Lo et al. 2023). Moreover, fluctuations in corticosterone across different phases of the estrous cycle can influence stress reactivity and the effectiveness of interventions such as exercise and sertraline (Atkinson and Waddell 1997; Hare et al. 2014; Rawat et al. 2018). Understanding these variations is therefore vital for designing tailored treatment strategies that account for the hormonal environment, ultimately enhancing behavioral outcomes in PTSD.

This study examines how exercise type and sertraline affect anxiety and social behavior in a female rat model of PTSD, focusing on corticosterone levels. Using a rodent PTSD model induced by a single prolonged stress protocol, we aim to explore treatment strategies that consider hormonal influences on behavioral outcomes. By correlating these fluctuations with response to exercise and pharmacotherapy, this research underscores the importance of integrating tailored interventions to enhance PTSD treatment, particularly for women.

## 2 Material and Methods

### 2.1 Animal Models

Sixty female adult Wistar rats, each weighing 242 ± 15 g, were obtained from the Laboratory Animal House at Urmia University, Urmia, Iran. The rats were housed individually on a 12‐h light/dark cycle, with lights on at 06:00 a.m., and were provided ad libitum access to food and water. Upon arrival, they were allowed a one‐week acclimation period before any experimental procedures commenced. The experiments were carried out between 08:00 and 18:00 h. The study obtained approval from the Medical Ethical Committee of Tabriz University (Approval No.: IR.TABRIZU.REC.1401.094) and was conducted in strict accordance with the National Institutes of Health (NIH) guidelines for the use of experimental animals.

### 2.2 Animal Groups and Timeline

**FIGURE 1:**
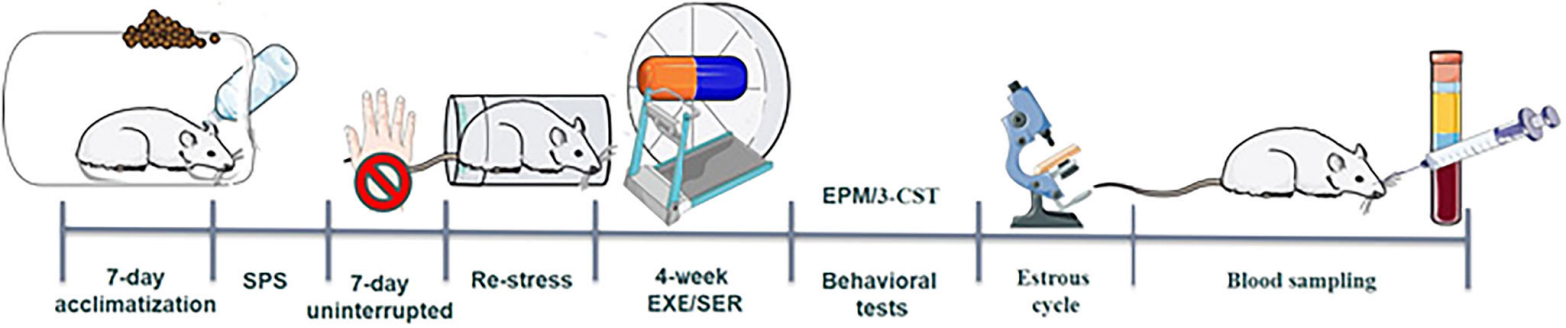
Experimental Time Scale. Abbreviations: 3‐CST = Three‐Chamber Social Test, EPM = elevated plus maze, EXE = exercise, SER = sertraline, SPS = single‐prolonged stress.

The present manuscript represents a subset analysis derived from a larger experimental dataset and specifically focuses on female SPS‐exposed rats in the metestrus and diestrus phases of the estrous cycle. Accordingly, only animals meeting these cycle‐stage criteria were included in the present analyses. Comparative analyses between control and SPS animals from the larger dataset have been reported separately (Sheikhzadeh Hesari et al. 2024). The experiment involved dividing animals into six distinct groups: SPS, SPS with involuntary exercise (SPS/IEXE), SPS with voluntary exercise (SPS/VEXE), SPS with sertraline (SPS/SER), SPS with sertraline and involuntary exercise (SPS/SER_IEXE), and SPS with sertraline and voluntary exercise (SPS/SER_VEXE). Following grouping, the animals underwent a series of behavioral tests and serum biomarker assessments. Figure 1 illustrates the timeline of the experimental procedures and treatment protocols, including sertraline administration, involuntary exercise, and voluntary exercise interventions (Figure 1).

### 2.3 Drug

In this study, sertraline hydrochloride was kindly supplied by the Faculty of Pharmacy at Tabriz University of Medical Sciences, Iran. Prior to drug administration, the daily water intake of each rat was recorded and used to calculate the corresponding dose in mg/kg body weight. The drug was delivered through the animals' drinking water at a concentration of 10 µg/mL daily for a duration of 4 weeks following the subsequent stress protocol. This method of oral administration via drinking water closely replicates the clinical usage of sertraline in humans and eliminates the stress associated with daily gavage handling (Dringenberg et al. 2014; Kryst et al. 2022).

### 2.4 Single‐Prolonged Stress

Utilizing the Single‐prolonged Stress (SPS) animal model of PTSD, rats underwent a sequence of experimental procedures. Initially, they were placed in a restraining tube with a diameter of 7 cm and a length of 14.5 cm, equipped with ventilated caps, where they remained for 2 h. After this period, the rats were subjected to a 20‐minute forced swimming session conducted in groups of five within a tall swim cylinder (36 cm diameter, 51 cm height), filled with water halfway up the cylinder. Following the swimming session, the rats underwent a 15‐min recovery period. They were subsequently exposed to diethyl ether for approximately 3 min. After a 7‐day undisturbed period in their home cages, the rats experienced a subsequent 10‐min restraint session to simulate PTSD reminders in the model (Uniyal et al. 2019; Verbitsky et al. 2020).

### 2.5 Voluntary Exercise With Running Wheels

Voluntary exercise was facilitated through individual cages equipped with 31 cm diameter running wheels monitored by reed switch sensors and electrical counters triggered by rotating magnets. The total number of revolutions, regardless of direction, was recorded at 10:00 a.m., aligned with the peak activity period of the rats during the dark cycle. Subjects had continuous 24‐h access to the running wheels over 4 weeks. Revolutions were converted to running distances based on wheel circumference. The mean ± SEM running distances per week were 1736.80 ± 275.4 meters in the voluntary exercise group and 2661.25 ± 456.9 meters in the voluntary exercise plus sertraline group.

### 2.6 Involuntary Exercise With Treadmill

Involuntary exercise was conducted using a treadmill to acclimate the subjects. A one‐week adaptation period involved light exercise, not considered part of the treatment period. The 10‐channel treadmill (Maze Router, Tabriz, Iran) was set at a 0% slope and operated 5 days a week from 11:30 a.m. to 12:00 p.m. during the light cycle. The exercise protocol consisted of 5 min at a speed of 10 m/min, followed by 20 min at 20 m/min, and concluded with 5 min at 10 m/min, resulting in a weekly running distance of 2500 ± 0 meters. After each session, the treadmill was wiped clean with 70% ethanol and air‐dried.

### 2.7 Experimental Design and Behavioral Assessment

#### 2.7.1 Elevated Plus Maze Test

The Elevated Plus Maze (EPM) test was utilized to evaluate anxiety‐like behaviors in a rodent model following a treatment phase (Carobrez and Bertoglio 2005). The EPM apparatus consisted of a plus‐shaped maze positioned 46 cm above the floor, featuring four arms (51 cm × 10 cm × 40 cm each) and a central platform (10 cm × 10 cm). Behavioral observations were captured using a video camera (Borj Sanat Azma Co., Tehran, Iran) placed 181 cm above the maze. Animals were individually placed in the central zone of the maze and monitored for 5 min. Key parameters assessed included reduced time spent in the open arms, frequency of entries into different maze arms, and increased time spent in closed arms, providing insights into anxiety levels post‐treatment (Lister 1987; Walf and Frye 2007). The percentage of time spent in the open arms was calculated as % open arm time = (time in open arms / total test duration) × 100, and the percentage of closed‐arm entries was calculated as: % closed‐arm entries = (closed‐arm entries / (open + closed arm entries)) × 100. The total traveled distance during the test was utilized to determine locomotor activity (Geis et al. 2012).

#### 2.7.2 Social Interaction Test

The three‐chamber Social Interaction Test (3‐CST) was employed to assess social behavior in rats (Nadler et al. 2004). The apparatus consisted of three chambers of equal size (30 × 32 cm and 39 cm high), separated by sliding doors. Each chamber featured an inverted empty black wire cup (10 cm diameter) with two weighted cups placed on top. The test comprised three phases.

In Phase 1, the adaptation phase, habituation to the center chamber involved placing the subject rat in the middle chamber with both sliding doors shut for 10 min. This allowed the rat to acclimate to the environment without any social stimuli.

Phase 2, the sociability phase, began with the introduction of a new, unfamiliar rat (stranger rat 1) into the right chamber of the cage. The left chamber contained an inverted empty wire cup. After opening the doors, the rats were given 10 min to explore the chambers. Sociability was assessed by manually measuring the time spent (in seconds) and the number of entrances into both the empty and stranger rat 1 chambers.

Phase 3, the social novelty phase, started with placing the subject rat back into the center chamber with both doorways closed. A different unfamiliar rat (stranger rat 2) was then placed in the previously empty chamber. Both sliding doors were opened simultaneously, allowing the subject rat to explore for 10 min. Social novelty was evaluated by manually measuring the time spent (in seconds) and the number of entrances into the chambers of stranger rat 1 and stranger rat 2 (Nadler et al. 2004, Yang et al. 2011).

### 2.8 Identification of Rat Estrous Cycle Stages

To determine the stage of the estrous cycle, vaginal smears were conducted on each rat. Each rat was positioned upright, and a 100 µL pipette containing sterile saline (0.9% NaCl) was introduced into the vaginal canal opening. Gentle flushing with 50 µL of saline was performed three to five times to collect the vaginal sample. The collected fluid was transferred to dry microscope slides and immediately examined under a light microscope (Olympus CH 2, Japan) at magnifications of 4X, 10X, and 40X.

The stages of the estrous cycle—proestrus, estrus, diestrus, and metestrus—were determined based on the types and proportions of cells observed in the collected samples (Marcondes, Bianchi et al. 2002; Caligioni 2009). The presence of cornified and nucleated epithelial cells indicated the metestrus phase, while leukocytes were indicative of both the metestrus and diestrus phases (Met/Die). Predominance of nucleated epithelial cells characterized proestrus, whereas anucleated cornified cells were characteristic of estrus (Pro/Est).

Vaginal secretions were collected from each rat after the final day of behavioral tests to determine their estrous cycle phase, mitigate additional stress and mechanical trauma, and account for hormonal influences on observed behavior (Becegato, Meurer et al. 2021). Based on previous studies, rats were classified into Pro/Est and Met/Die subgroups, with rats randomly selected for analysis, including *n* = 5 from each Met/Die group (Guillén‐Ruiz et al. 2021; Rodríguez‐Landa, Guillén‐Ruiz et al. 2021).

### 2.9 Blood Collection and Enzyme‐Linked Immunosorbent Assay

Blood samples for enzyme‐linked immunosorbent assay (ELISA) were obtained from rats one day after identifying their estrous cycle stage. The rats were anesthetized using a standard mixture of ketamine (50 mg/kg) and xylazine (5 mg/kg) (Sohroforouzani, Shakerian et al. 2022), administered intraperitoneally between 8:00 a.m. and 2:00 p.m. Samples (3 mL per rat) were collected from the eye sinus and centrifuged at 12,000 rpm for 10 min to obtain serum, which was then stored at −80°C until further analysis. Serum corticosterone concentrations were quantified using a rat‐specific ELISA kit supplied by ZellBio GmbH (Germany). All procedures followed the manufacturer's guidelines. The assay demonstrated high reliability, with intra‐ and inter‐assay coefficients of variation below 4.1% and 6.4%, respectively. The detection limit of the kit was less than 1.6 nmol/L.

### 2.10 Statistical Analysis

Data were analyzed using IBM SPSS (v26.0) and GraphPad Prism (v10.2.3), with results expressed as means ± SEM. Normality of the data distribution was assessed via the Shapiro–Wilk test.

Statistical analyses were primarily conducted using two‐way ANOVA, with exercise and sertraline treatment as the independent factors, followed by Bonferroni's post‐hoc tests for pairwise comparisons. To account for potential confounding effects of locomotor activity on anxiety‐like behavior, a two‐way ANCOVA was applied to the EPM data, utilizing total distance traveled as a covariate.

Relationships between variables were evaluated using Pearson correlation coefficients. To account for the increased risk of Type I errors due to multiple correlations, *p*‐values were adjusted using the Benjamini‐Hochberg procedure with the False Discovery Rate (FDR) set at 5% (*Q* = 0.05). For all other statistical tests, significance was set at *p* < 0.05.

## 3 Results

### 3.1 Elevated Plus Maze Test

#### 3.1.1 Time Spent in the Open Arms

To evaluate anxiety‐like behavior while controlling for locomotor activity, a two‐way ANCOVA was conducted with Total Distance Moved as a covariate. The analysis showed that the covariate did not significantly influence the time spent in open arms (*F* (1, 23) = 1.487, *p* = 0.235), confirming that the observed differences in anxiety levels were not confounded by general locomotion. After adjusting for the covariate, significant main effects were found for both Exercise (*F* (2, 23) = 16.981, *p* < 0.001) and Sertraline (*F* (1, 23) = 14.040, *p* = 0.001). However, the interaction between Exercise and Sertraline was not statistically significant (*F* (2, 23) = 0.770, *p* = 0.475), suggesting that the effect of Sertraline was consistent across all exercise conditions.

Post‐hoc comparisons revealed that the SPS+IEXE group (M = 27.599) spent significantly more time in the open arms compared to both the SPS (No EXE) group (M = 15.256, *p* < 0.001) and the SPS+VEXE group (M = 18.208, *p* = 0.001). In contrast, there was no significant difference between the SPS+VEXE and SPS (No EXE) groups (*p* = 0.675). Regarding the treatment effect, animals receiving Sertraline (SER) spent significantly more time in the open arms (M = 26.345) compared to the non‐sertraline group (N0_SER) (M = 14.364, *p* = 0.001).

**FIGURE 2:**
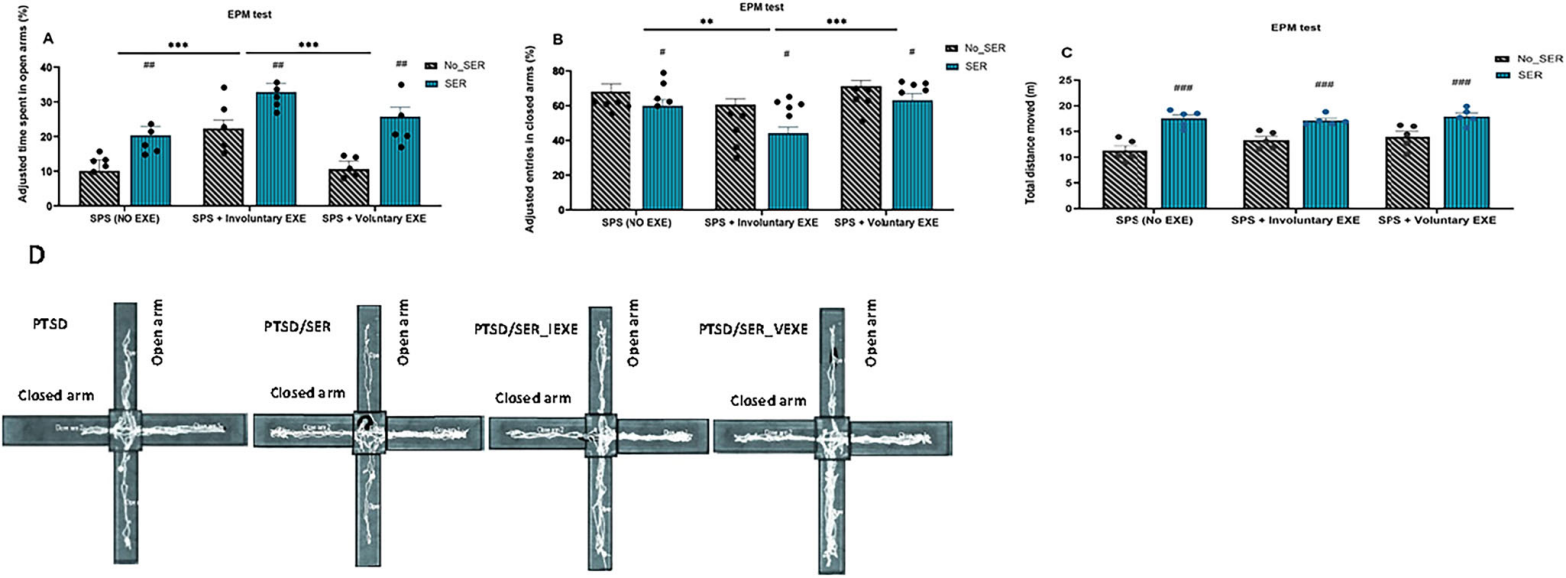
Effects of sertraline and exercise on anxiety‐like behavior in the Elevated Plus Maze (EPM) test. (A) Adjusted percentage of time spent in the open arms, (B) Adjusted percentage of entries into the closed arms; both parameters were analyzed using ANCOVA with “total distance moved” as the covariate to account for differences in locomotor activity, (C) Total distance moved (m) during the test, and (D) representative video tracking images in the EPM. Data are presented as mean ± SEM. Significant differences between exercise groups are indicated by asterisks (***p < 0.001 and **p < 0.01). Significant effects of sertraline treatment within each exercise condition are indicated by hashtags (###p < 0.001, ##p < 0.01, and #p < 0.05).

These results indicate that while Sertraline effectively reduces anxiety‐like behavior, involuntary exercise (IEXE) provides a superior anxiolytic effect compared to voluntary exercise or no exercise in the SPS model, independent of the animal's total movement (Figure 2A).

#### 3.1.2 Number of Closed‐Arm Entries

To account for general locomotor activity, a two‐way ANCOVA was conducted for the number of entries into the closed arms, using Total Distance Moved as a covariate. The analysis showed that the covariate did not have a significant effect on the number of closed‐arm entries (*F* (1, 23) = 0.017, *p* = 0.896), suggesting that the differences in entry counts were independent of the total distance traveled. After adjusting for the covariate, significant main effects were observed for both Exercise (*F* (2, 23) = 12.562, *p* < 0.001) and Sertraline (*F* (1, 23) = 5.872, *p* = 0.024). However, the interaction between Groups and Treatment was not statistically significant (*F* (2, 23) = 1.118, *p* = 0.344).

Post‐hoc comparisons revealed that the SPS+IEXE group (M = 52.436) had significantly fewer closed‐arm entries compared to both the SPS (No EXE) group (M = 64.064, *p* = 0.004) and the SPS+VEXE group (M = 67.261, *p* < 0.001). No significant difference was observed between the SPS+VEXE and SPS (No EXE) groups (*p* = 1.000). Regarding the treatment effect, animals receiving Sertraline (SER) showed a significantly lower number of closed‐arm entries (M = 55.791) compared to the non‐sertraline (N0_SER) group (M = 66.717, *p* = 0.024).

These results indicate that when overall locomotor activity is controlled, both Sertraline and involuntary exercise (IEXE) significantly reduce the number of closed‐arm entries, which may suggest a reduction in avoidance behavior or adjusted exploratory activity. Notably, IEXE demonstrated a more pronounced effect compared to voluntary exercise, which did not differ from the SPS group (Figure 2B).

#### 3.1.3 Total Distance Traveled

To examine general locomotor activity, a two‐way ANOVA was conducted. The analysis revealed a significant main effect of Sertraline treatment (*F* (1, 24) = 51.870, *p* < 0.001), while no significant effect was found for Exercise (*F* (2, 24) = 1.863, *p* = 0.177) or the Interaction between Exercise and Treatment (*F* (2, 24) = 1.499, *p* = 0.244).

Post‐hoc comparisons and estimated marginal means confirmed that Sertraline‐treated animals displayed significantly higher locomotor activity (M = 17.519) compared to the non‐sertraline‐treated (N0_SER) group (M = 12.845, *p* < 0.001).

These results indicate that Sertraline primarily enhanced locomotor activity in the SPS model, regardless of the exercise condition, while exercise itself (voluntary or involuntary) did not significantly alter the total distance traveled compared to the SPS group (Figure 2C).

### 3.2 Social Interaction Test

#### 3.2.1 Sociability

To evaluate sociability, a two‐way ANOVA was performed on the number of entries into the chamber containing the stranger rat. The analysis revealed a significant main effect of Exercise (*F* (2, 24) = 17.355, *p* < 0.001) and a significant Interaction between Exercise and Sertraline (*F* (2, 24) = 4.897, *p* = 0.016). However, the main effect of Sertraline treatment alone did not reach statistical significance (*F* (1, 24) = 2.798, *p* = 0.107).

Post‐hoc comparisons revealed that the SPS+IEXE group (M = 10.800) showed significantly higher sociability scores compared to both the SPS (No EXE) group (M = 6.200, *p* < 0.001) and the SPS+VEXE group (M = 8.400, *p* = 0.029). Furthermore, the SPS+VEXE group also showed significantly higher sociability compared to the SPS (No EXE) group (*p* = 0.029). Regarding the treatment effect, although animals receiving Sertraline (SER) showed a higher mean (M = 9.000) compared to the non‐sertraline (N0_SER) group (M = 7.933), this difference did not reach statistical significance (*p* = 0.107).

**FIGURE 3:**
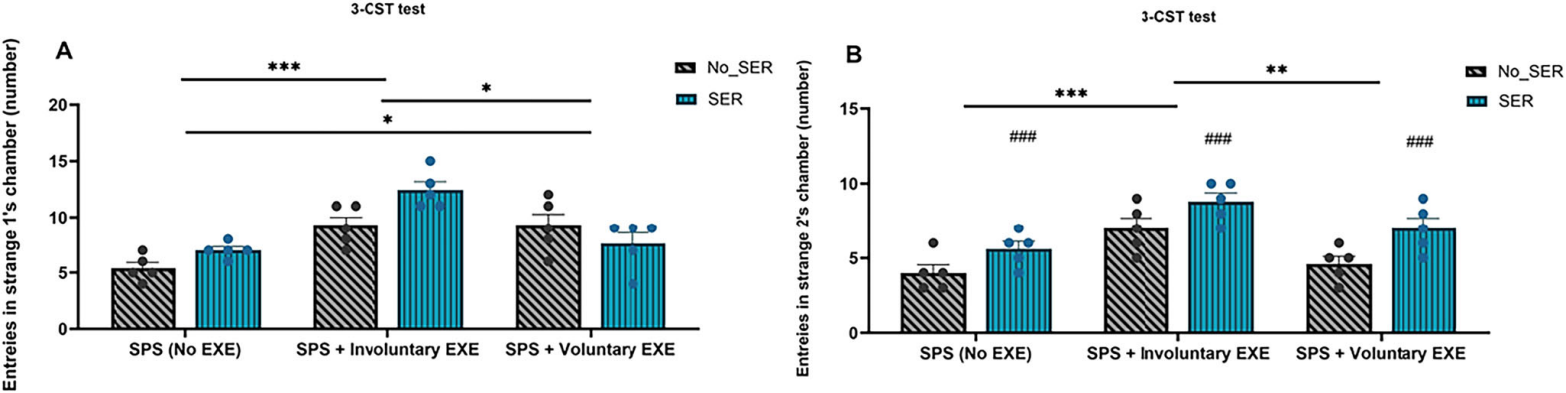
Effects of sertraline and exercise on social behavior in the Three‐Chamber Social Test (3‐CST). (A) Number of entries into the chamber containing stranger 1 and (B) number of entries into the chamber containing stranger 2. Data are presented as mean ± SEM. Significant differences between groups (indicated by comparison bars) are denoted by asterisks: and ***p < 0.001, **p < 0.05, and *p < 0.01. Significant effects of sertraline treatment within each exercise condition are indicated by hashtags (###p < 0.001).

These results indicate that involuntary exercise (IEXE) significantly enhances sociability. Notably, IEXE demonstrated a more pronounced effect compared to voluntary exercise (VEXE), although both types of exercise were effective in increasing social exploration compared to the SPS group (Figure 3A).

#### 3.2.2 Social Novelty

To assess preference for social novelty, a two‐way ANOVA was conducted on the number of entries into the chamber containing the unfamiliar new rat (Stranger 2). The analysis revealed significant main effects for both Exercise (*F* (2, 24) = 13.914, *p* < 0.001) and Sertraline (*F* (1, 24) = 15.568, *p* < 0.001). However, the interaction between Exercise and Sertraline was not statistically significant (*F* (2, 24) = 0.240, *p* = 0.788), suggesting that the treatment effect was consistent across different exercise conditions.

Post‐hoc comparisons revealed that the SPS+IEXE group (M = 7.900) showed significantly higher social novelty scores compared to both the SPS (No EXE) group (M = 4.800, *p* < 0.001) and the SPS+VEXE group (M = 5.800, *p* = 0.006). However, the SPS+VEXE group did not show a statistically significant difference in social novelty compared to the SPS (No EXE) group (*p* = 0.326). Regarding the sertraline effect, animals receiving Sertraline (SER) showed a significantly higher mean (M = 7.133) compared to the non‐sertraline (N0_SER) group (M = 5.200, *p* < 0.001).

These results indicate that involuntary exercise (IEXE) significantly enhances social novelty, demonstrating a more pronounced effect compared to voluntary exercise (VEXE). While Sertraline treatment was effective across all groups, IEXE was the only exercise modality that significantly increased social novelty compared to the SPS group in this study (Figure 3B).

### 3.3 Corticosterone

To evaluate the physiological stress response, a two‐way ANOVA was performed on serum corticosterone levels. The analysis revealed a significant main effect of Sertraline treatment (*F* (1, 24) = 54.219, *p* < 0.001) and a highly significant Interaction between Exercise and Sertraline (*F* (2, 24) = 21.436, *p* < 0.001). The main effect of Exercise alone did not reach statistical significance (*F* (2, 24) = 0.440, *p* = 0.649).

Post hoc analysis revealed that in SPS animals, sertraline treatment significantly reduced corticosterone levels compared to untreated SPS rats (*p* < 0.05). Notably, the combination of sertraline with involuntary exercise (IEXE) resulted in a more pronounced decrease in corticosterone, showing a highly significant reduction compared to the untreated SPS group (*p* < 0.001).

**FIGURE 4:**
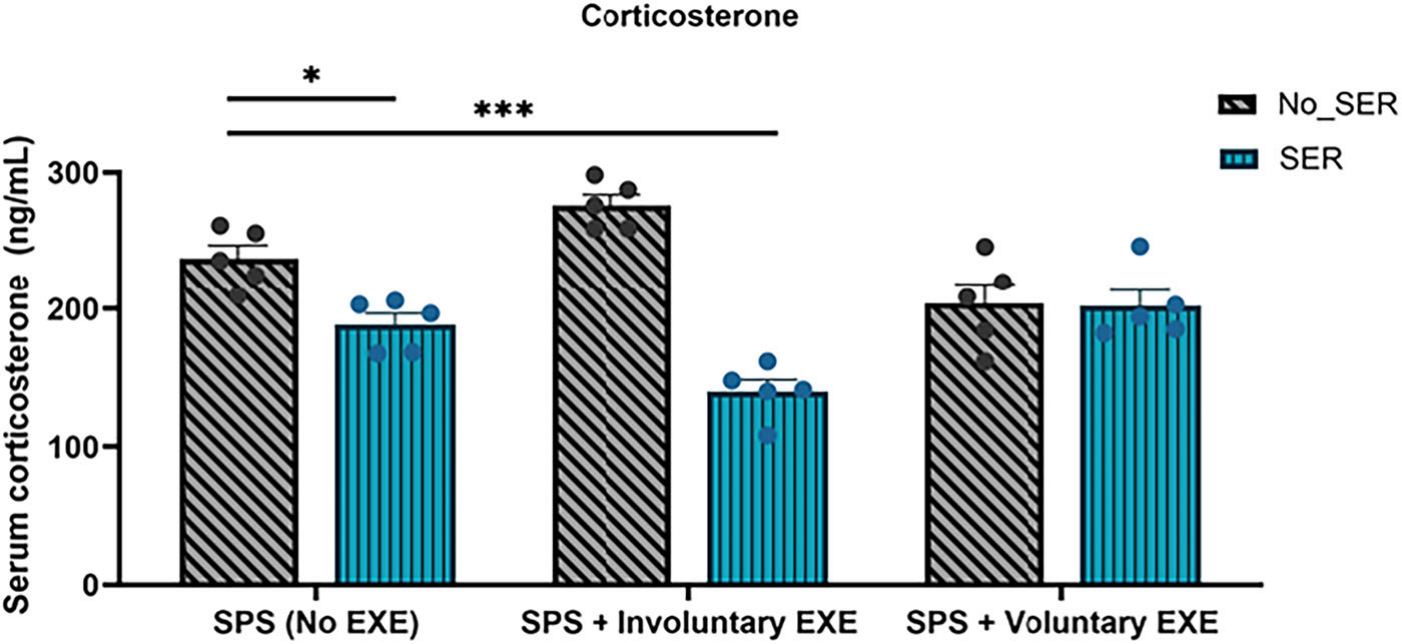
Effects of sertraline and exercise on serum cortisol levels. Data are presented as mean ± SEM. Significant differences between groups (indicated by comparison bars) are denoted by asterisks: ***p < 0.001 and *p < 0.05.

These results indicate that while Sertraline treatment effectively reduces physiological stress across groups, its impact is significantly enhanced when combined with involuntary exercise (IEXE). This synergy is reflected in the highly significant interaction between the two factors, where the involuntary exercise and sertraline combination led to the most pronounced reduction in serum corticosterone levels compared to the untreated SPS group (Figure 4).

### 3.4 Correlation Analysis

Pearson's correlation analysis was conducted to explore the associations between behavioral measures and serum corticosterone levels. To account for multiple comparisons and the potential inflation of Type I error, *p*‐values were adjusted using the Benjamini–Hochberg false discovery rate (FDR) procedure (*Q* = 5%).

**FIGURE 5:**
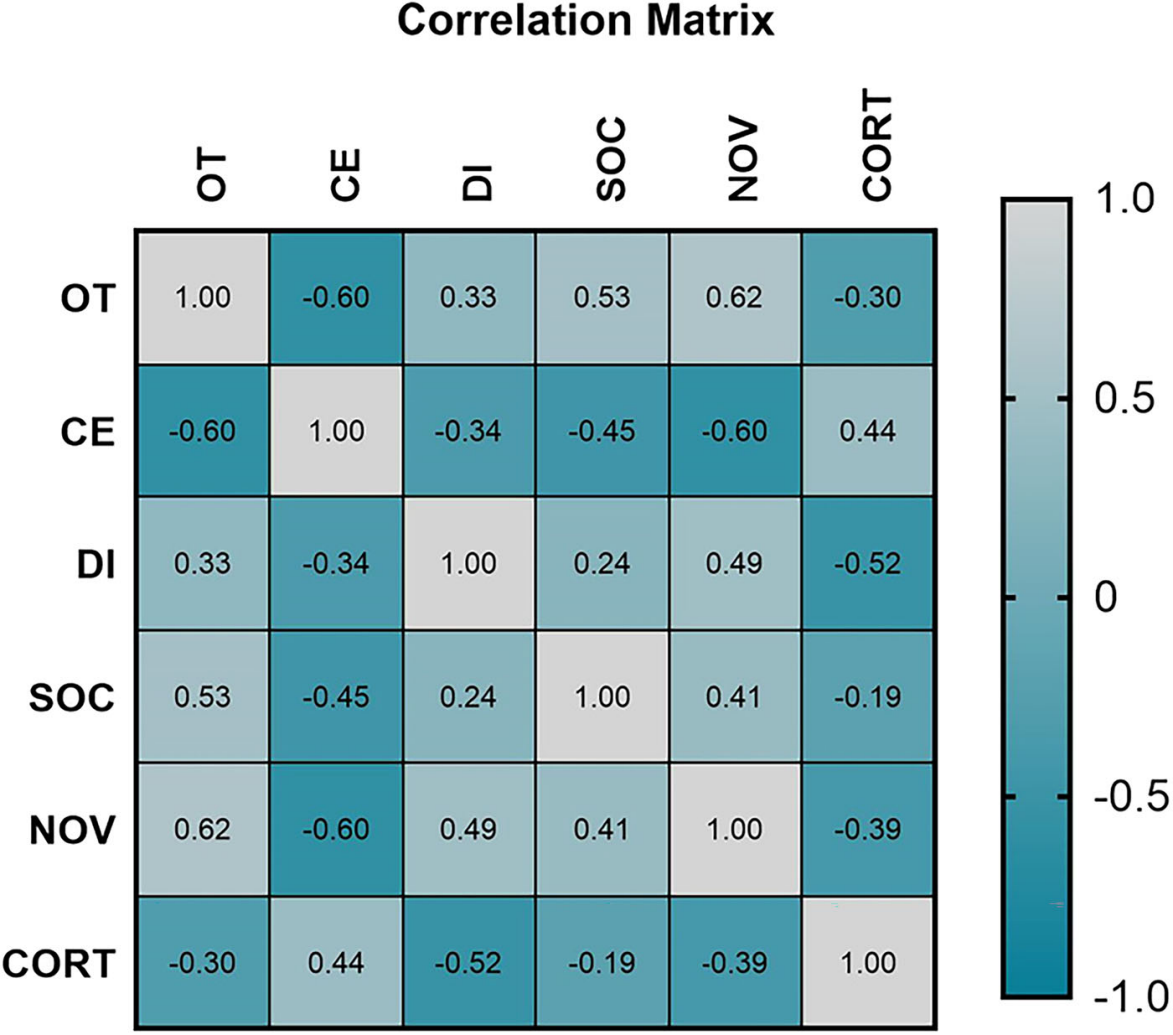
Pearson correlation matrix between behavioral parameters and plasma cortisol levels. Values represent correlation coefficients (*r*). Color scale indicates the strength and direction of the correlation (from −1 to +1). Abbreviations: CE = closed arm entries (EPM), CORT = serum cortisol, DI = distance traveled (EPM), OT = open arm time (EPM), SOC = sociability (3‐CST), NOV = social novelty preference (3‐CST),.

The analysis revealed several significant associations (Figure 5). Open arm time (OT) in the EPM test showed moderate to strong positive correlations with both sociability (SOC; *r* = 0.53, *q* = 0.010) and social novelty (NOV; *r* = 0.62, *q* = 0.002). Conversely, a significant negative correlation was observed between OT and closed‐arm entries (CE; *r* = −0.60, *q* = 0.002). These findings suggest that reduced anxiety‐like behavior is significantly associated with improved social interaction and a higher preference for social novelty.

In contrast, serum corticosterone (CORT) levels displayed a significant negative correlation with distance traveled (DI; *r* = −0.52, *q* = 0.010) and social novelty (NOV; *r* = −0.39, *q* = 0.049). Additionally, a significant positive correlation was found between CORT and closed‐arm entries (CE; *r* = 0.44, *q* = 0.028). While a negative trend was initially observed between corticosterone and open arm time (OT), this association did not remain statistically significant after FDR correction (*r* = −0.29, *q* = 0.128).

Together, these correlations indicate that anxiolytic‐like behavioral outcomes, characterized by greater open arm exploration, higher sociability, and increased novelty preference, are significantly associated with lower physiological stress markers. These findings suggest a consistent relationship between behavioral and biochemical indices of stress and anxiety.

## 4 Discussion

The present study explored how voluntary and involuntary exercise, in combination with sertraline, affect anxiety and social behavior in female rats in a PTSD model. The results demonstrate significant differences in the efficacy of the interventions, with involuntary exercise showing more pronounced anxiolytic effects compared to voluntary exercise. Both sertraline treatment and involuntary exercise were associated with reduced anxiety‐like behavior in the EPM test. These findings have important implications for PTSD treatment, particularly in females, given the higher prevalence of PTSD and anxiety disorders in women (Tolin and Foa 2008; Kessler 2005).

These results are consistent with clinical data showing that sertraline reduces anxiety‐related symptoms in patients with PTSD (Brady, Pearlstein et al. 2000) and that structured exercise programs improve anxiety and stress management in affected individuals (Khameslo et al. 2023). Our results are consistent with previous studies, in which female rats with PTSD treated with fluoxetine, another SSRI, and moderate treadmill exercise, showed improved outcomes in anxiety (Shafia et al. 2023). Specifically, sertraline has been shown to reduce anxiety by increasing serotonin levels in the brain, which helps regulate mood and stress responses (Heesbeen, van Kampen et al. 2024). Our findings agree with previous studies that have demonstrated involuntary exercise appears to exert a robust anxiolytic effect when paired with sertraline in a PTSD animal model, possibly due to the controlled nature of the intervention, ensuring consistent physical activity levels (Zhang, Xue et al. 2020). According to some studies, the effects of exercise on anxiety‐like behaviors may vary depending on the type, intensity, and duration of the physical activity (Alipour et al. 2022, Hwang and Kim 2024). A study suggests that involuntary exercise may help mitigate stress and anxiety in rats by reducing variability in physical activity levels, which could be attributed to the structured and consistent nature of involuntary exercise (Ercan, Bulmus, et al. 2023). Notably, clinical studies similarly indicate that structured exercise programs, compared with unstructured or irregular physical activity, are associated with more reliable improvements in physiological and psychosocial outcomes, supporting the translational relevance of structured exercise paradigms (Sharma, Subramanian et al. 2017; Keating et al. 2019). Moreover, the hormonal fluctuations associated with the estrous cycle may have influenced the observed differences in anxiety‐related behaviors, as previous research has shown that female rats exhibit varying stress responses across different phases of the cycle (Miller et al. 2021). In the present study, we attempted to control for hormonal influences by analyzing data from rats in the metestrus and diestrus phases.

Involuntary exercise, particularly when combined with sertraline, was associated with reduced corticosterone levels, while exercise, especially involuntary exercise, significantly enhanced social interaction in the 3‐CST compared to untreated PTSD model female rats. Previous studies showed that moderate treadmill exercise reduced elevated corticosterone in ovariectomized rats subjected to a PTSD model, showing exercise can lower stress hormones even under ovarian hormone deficiency (Yakhkeshi, Roshani et al. 2022). There is evidence that involuntary exercise, when combined with sertraline, reduced serum corticosterone levels in aged female rats (Gokdemir, C. et al. 2020). Exercise has been shown to have a wide range of effects, including improvements in social interaction. In line with this, a study by Nguyen et al. (2013) reported for the first time that 30 days of low‐intensity treadmill exercise led to increased social interaction in rats, suggesting that exercise can positively influence social behavior (Nguyen et al. 2013). Clinical studies suggest that structured and regular exercise programs are more effective in social interaction, communication, and cooperation (Zhao and Chen 2018). In addition to exercise, the effects of sertraline on social interaction have also been examined in several studies. One study found that sertraline can reverse social withdrawal induced by ketamine in male mice, further supporting its potential to improve social interaction (Onaolapo, Paul, et al. 2017).

Furthermore, sertraline increased the running distance in voluntary running wheels, suggesting a general increase in activity levels. The increase in total distance traveled could be attributed to several factors. While sertraline primarily targets mood disorders by increasing the availability of serotonin (Wong et al. 1995), its effects can also include heightened general activity and exploration (Cryan and Mombereau 2004; Kott, Mooney‐Leber, et al. 2019). In line with our study, several studies have reported that sertraline improves locomotor activity and attenuates motor performance deficits in male rats with Huntington's‐like symptoms (Gill, Jamwal et al. 2017), as well as in healthy male and female rats (Yildirim, Erol et al. 2012).

In addition to the main behavioral and biochemical findings, correlation analyses provided further support for the convergent validity of these outcomes after controlling for multiple comparisons. In adult female Wistar rats, reduced anxiety‐like behavior was significantly associated with increased sociability and a greater preference for social novelty. Furthermore, elevated serum corticosterone levels were significantly associated with reduced exploratory activity (distance traveled) and lower social novelty, along with higher closed‐arm entries. Consistent with these results, a recent study in socially isolated male Wistar rats reported that higher corticosterone levels were associated with increased anxiety‐like behavior, supporting the link between stress hormones and behavioral responses across sexes (Ngala, Hemmings, et al. 2025). In contrast, another study using adult Heterogeneous Stock rats and a modified social interaction test reported weaker correlations between social and anxiety‐like behaviors (Wang et al. 2018).

The study's findings on involuntary exercise combined with sertraline may have broader implications for other anxiety‐ and mood‐related disorders, including generalized anxiety disorder, social anxiety disorder, and major depressive disorder. Although the present findings support the potential therapeutic value of structured exercise combined with sertraline in a female PTSD model, caution is warranted when extrapolating these results beyond the current experimental conditions. Future studies should determine whether the observed behavioral and corticosterone‐related effects are reproducible across different phases of the estrous cycle and in male subjects. In addition, further investigation is required to clarify the neural and molecular mechanisms underlying the interactive effects of involuntary exercise and sertraline on stress‐ and anxiety‐related behaviors.

## 5 Limitations

Several constraints in the present study warrant consideration. Primarily, the exclusive use of female rats precludes a direct comparison between sexes. Furthermore, to preserve the integrity of our structured exercise paradigm, voluntary activity levels were not matched to the treadmill group. Additionally, due to the limited number of animals and the uneven distribution across estrous stages, analyses were restricted to rats in the metestrus and diestrus phases to maintain statistical feasibility. As a single‐point assessment, these results provide only a limited approximation of the hormonal environment during stress and testing. Consequently, estrous cycle‐related data were not included as a primary analytical factor, and future studies with larger sample sizes are required to evaluate the influence of specific estrous phases.

## 6 Conclusion

In conclusion, involuntary exercise demonstrated the most pronounced beneficial effects on anxiety‐like behaviors and social outcomes in female rats subjected to the SPS model. Sertraline treatment independently contributed to improvements in behavioral and physiological measures. The combination of involuntary exercise and sertraline produced the greatest reduction in corticosterone levels, indicating a potential synergistic effect on physiological stress responses. These findings highlight the value of structured physical activity alongside pharmacological treatment in PTSD models, although further research is required to clarify the underlying mechanisms and their translational relevance.

## Author Contributions

**Sarieh Ebrahimiasl**: investigation, writing – original draft, and formal analysis. **Farzam Sheikhzadeh Hesari**: project administration, methodology, writing – review and editing.**Mohammad Reza Khansari**: writing – review and editing. **Afshin Dana**: methodology. **Khadijeh Imani Minaabad**: investigation.

## Funding

The authors have nothing to report.

## Ethics Statement

All animal experiments that were described in this study were followed according to the National Institutes of Health Guide for the Care and Use of Laboratory Animals (Council 2010). The study was approved by the Medical Ethical Committee of the University of Tabriz (Approval No.: IR.TABRIZU.REC.1401.094).

## Consent

All authors consent for publication.

## Conflicts of Interest

The authors declare no conflicts of interest.

## Data Availability

The data and materials that support the findings of this study are available from the corresponding author upon reasonable request.
